# Leveraging Technology to Increase Behavioral Health Services Access for Youth in the Juvenile Justice and Child Welfare Systems: a Cross-systems Collaboration Model

**DOI:** 10.1007/s11414-022-09808-1

**Published:** 2022-07-14

**Authors:** Marina Tolou-Shams, Evan D. Holloway, Catalina Ordorica, Juliet Yonek, Johanna B. Folk, Emily F. Dauria, Kristiana Lehn, Ifunanya Ezimora, Honorable Monica F. Wiley

**Affiliations:** 1 Department of Psychiatry and Behavioral Sciences, Zuckerberg San Francisco General, University of California, 1001 Potrero Ave, Bldg. 5, 7M, San Francisco, CA 94110 USA; 2 Department of Behavioral and Community Health Sciences, University of Pittsburgh, Pittsburgh, PA USA; 3 San Francisco Unified Family Court, San Francisco, USA

## Abstract

Behavioral health services access for justice- and child welfare-involved youth is limited despite significant need. Structural interventions to address limited access are nascent. Technology can advance access, but few interventions focus on system-impacted youth and their mental health needs and challenges. This article describes the development, process, and initial outcomes of the Youth Justice and Family Well-Being Technology Collaborative (JTC) that was formed to leverage technology within and across public health and justice-related systems to promote increased behavioral health services access. Cross-system considerations are identified for public health, court, and other key stakeholders to successfully integrate technology into practice to expand access to these critical services.

## Introduction

Youth in contact with and impacted by the juvenile justice and child welfare systems (herein referred to as system-impacted youth) have high rates of substance use and mental health needs but experience significant disparities in behavioral health services access and engagement relative to youth who are not system-impacted. Among youth at first juvenile court contact (14.5 years old), 50% endorse cannabis use and 30% report clinically significant mental health needs.^1^ Of the 50% not using cannabis at first contact, 18% initiate cannabis use within the subsequent 12 months.^2^ Experiencing multiple adverse childhood experiences (ACEs), such as parental incarceration, domestic violence exposure, and abuse, is also common by first system contact, and abuse ACEs predict alcohol use and posttraumatic stress symptoms 12 months after initial court contact.^3^ Yet surprisingly, only 8–16% of youth with legal system contact receive behavioral health services.^4,5^ Factors associated with disparities in service access are complex and include individual, family,^6,7^ neighborhood,^8^ and structural barriers associated with racism, poverty, and other macro-level influences.^6,9,10^ For example, a national survey of juvenile justice community supervision agencies and associated behavioral health provider agencies found that 33% of these systems provide youth substance use prevention services;^11^ this is a staggeringly low proportion of services availability relative to need. Even when behavioral health needs are identified, other barriers prevent youth from finding, accessing, and engaging in services. One significant barrier stems from siloed serving systems, which hampers the necessary cross-system collaboration that can improve youth access to community-based care.^12–14^ For example, the juvenile dependency court legally oversees the care of all foster care youth. It is responsible for the safety, health, and well-being of these youth in conjunction with *multiple* stakeholders in *numerous* systems (e.g., child welfare workers, attorneys, schools, caregivers, behavioral health providers). Implementation of local change teams^15,16^ and development of cross-system collaborative tools^17^ are examples of promising, empirically supported approaches to increasing access to behavioral health services for system-impacted youth and families. Technological advances also hold promise to support increased behavioral health services access; yet, there has been minimal empirical investigation of the use of technology for this purpose and this population of youth, at any level.^18^

In 2020, the Youth Justice and Family Well-Being Technology Collaborative (JTC) was formed to identify and resolve juvenile justice and child welfare system-level barriers to youth behavioral healthcare services access and utilization, specifically using technology. There are three goals of this paper: first, to describe the development, guiding frameworks, and implementation of the JTC model and approach; second, to outline the JTC composition and logistical processes; and third, to present preliminary descriptive outcomes, lessons learned, and suggest next steps for the fields of public health and juvenile justice to consider for cross-system collaborative approaches to advance behavioral health equity through technology for system-impacted youth.

### Development of the JTC

The JTC is a collaborative academic-community partnership led by academic and judicial co-chairs and composed of stakeholders from six system categories serving system-impacted youth (i.e., behavioral health, diversion, legal, child welfare, school, juvenile probation). The JTC was originally formed within the context of a National Institute on Drug Abuse (NIDA) funded pilot efficacy trial of a youth and caregiver text messaging intervention to enhance substance use treatment engagement among youth on probation^19^. The original scientific aim of the collaborative was to understand systems’ perspectives on how such a text messaging intervention might be adopted and implemented by probation, behavioral health services providers, and/or child welfare serving systems (for dually-involved youth). The onset of COVID-19 and shelter-in-place mandates in March 2020, however, led to stakeholders’ request for more frequent meetings that would leverage the JTC as a vehicle for general cross-system discussions and rapid planning related to deploying telehealth interventions for court, probation, child welfare, and behavioral health services for system-impacted youth and families. Using a community-participatory research approach,^20^ the authors rapidly expanded the JTC’s aims to focus on system-level barriers to and facilitators of broader technology use for behavioral health cross-system service engagement and care coordination.

### Frameworks

The JTC relies on the Juvenile Justice Behavioral Health Services Cascade of Care (Cascade)^21^ and the Telehealth Capacity Assessment Tool (TCAT) and Strengthening Plan Model^22^ as frameworks to organize goals and projects.

#### Behavioral health cascade for system-impacted families

Modeled after the HIV Cascade of Care originally developed to improve implementation and uptake of healthcare services for those living with HIV,^21^ the Cascade framework was adapted by the Juvenile Justice Translational Research on Interventions for Adolescents in the Legal System (JJ-TRIALS; a cooperative research initiative funded by NIDA)^16^ to provide a framework to study gaps in implementation and increased substance use services for community-supervised youth (e.g., diversion, probation).

#### TCAT and capacity strengthening plan model

Developed by the National Frontier and Rural Addiction Technology and Transfer Center, the TCAT was designed to assist behavioral health organizations to assess their readiness to adopt telehealth technology to increase behavioral health services access. The TCAT highlights six domains (see Measures) that require assessment of their interplay to identify organizational capacity building needs and to develop a Capacity Strengthening Plan to successfully use telehealth technology. The TCAT can also be used to monitor and evaluate the impact of organizational capacity building over time. JTC stakeholders broadened the application of the TCAT framework to (1) incorporate any technology-based interventions and not just telehealth (e.g., text messaging, digital health mobile apps) and (2) expand TCAT data collection to other systems beyond behavioral health to examine technology services readiness and capacity within *and* across multiple JTC systems and organizations (e.g., courts, child welfare, probation). A TCAT Capacity Strengthening Plan is dynamic and intended to be iteratively revised according to plan progress. The templated plan includes (1) identified TCAT domain and domain subcomponent gaps (e.g., within the domain of technology, the subcomponent of technology infrastructure is identified as a gap), (2) listing internal and external resources needed to address the gap, (3) specific planned actions to close the gap, (4) priority determination (i.e., to address multiple gaps within and across domains), (4) assigning stakeholder(s) responsibility for planned actions, and (5) result description.

### JTC structure and implementation

Based on initial meetings guided by the Cascade and TCAT frameworks, the JTC formed subcommittees to allow for simultaneous progress on multiple projects. Subcommittees aligned with key “stuck” points in the Cascade (i.e., Initial Access and Engagement subcommittees). The JTC also identified a need for a Data Gathering/Sharing subcommittee to support the development and implementation of data-driven approaches to address identified gaps. Below are two examples of subcommittee identified gaps and associated capacity strengthening plans.

The Initial Access subcommittee focuses on the TCAT domain of Workforce Capacity and aims to use technology to build clinical workforce capacity. Stakeholders identified the lack of community-based child mental health providers with empirically supported substance use intervention training as a workforce gap to address. As part of their Capacity Strengthening Plan, the group is leveraging an existing tele-mentoring model^23^ at the University of California, San Francisco (UCSF; Project ECHO Juvenile Justice Behavioral Health) to provide ongoing telehealth training and case consultation on adolescent substance use assessment and intervention to behavioral health providers serving system-impacted youth; ECHO is an internationally implemented learning, mentoring, and peer support model to improve health outcomes of underserved populations.^23^ The Initial Access subcommittee co-developed an ECHO curriculum on trauma-responsive adolescent substance use assessment and intervention that has been successfully implemented with child behavioral health providers in Northern California who serve system-impacted youth.

The Data Gathering/Sharing subcommittee focuses on the TCAT domain of Technology. Available data regarding youth and family technology access was identified as a gap particularly because personnel (e.g., attorneys, behavioral health providers) continue to encounter barriers to technology access for youth and families under probation supervision. The Data Gathering/Sharing subcommittee developed a two-question screener to assess at intake to the county juvenile justice system, whether each youth had access to a technology device (e.g., laptop) and internet access. Stakeholders wanted to develop standardized items that would be incorporated into each juvenile legal system’s intake procedures so that courts, probation, public defender, and district attorney could systematically advocate for youth technology access, as needed. The Capacity Strengthening Plan also includes working closely with juvenile legal stakeholders to allow other system stakeholders access to these data so that they may collaborate in reducing barriers to technology access for individual youth and families in need.

## Methods

### Procedures

The JTC consists of 20 key stakeholders working in the court which include judges, attorneys, juvenile probation, diversion, child welfare, public health, educational, behavioral health provider, and academic research as administrators, behavioral health clinicians, policymakers, and data scientists. Stakeholders identified primarily as female (66.67%), average age of 45.78 (*SD* = 8.29), have been in their position for an average of 11 years (*SD* = 12.81), and serve a range of justice-impacted youth from pre-adjudicated non-detained (55.6%) to youth in placement (83.3%).

This study was approved by the University of California San Francisco’s IRB as a minimal risk study. Eighteen meetings have been held between January 2020 and September 2021. Stakeholders provided consent during an individual Zoom meeting. Among the six categories of providers (i.e., behavioral health, diversion, legal, child welfare, school, juvenile probation), the authors have had at least one stakeholder from each category at nine of 18 meetings with at least four of six categories represented at every meeting.

Each meeting’s agenda include (a) check-in, (b) prior meeting updates (e.g., policy changes, research project updates, action items), (c) a didactic presentation (e.g., participating system presenting service access data), and (d) subcommittee goals. Co-chairs review the agenda and share with JTC members 1 week prior to the meeting to elicit any additional agenda items. The research team provides note-takers for general (*n* = 2) and subcommittee discussions (*n* = 3). The research team separately debriefs after each meeting to share reflections and prioritize action items. The research project coordinator compiles and stores the raw notes for internal data analysis and creates a one-page summary to be shared with JTC members. Subcommittee co-chairs email members with actions items related to the cross-system Capacity Strengthening Plan.

### Measures

The authors used a mixed-methods approach using themes identified from JTC meeting notes and participant TCAT data across 3 timepoints, 6 months apart over an 18-month period (May 2020, November 2020, May 2021) to identify gaps and strengths in cross-system capacity to use technology to increase behavioral health services access.

#### Quantitative — telehealth capacity assessment tool

The TCAT is a 68-item self-report measure designed for stakeholders to plan, design, and monitor implementation of telehealth technology services.^22^ The six TCAT domains and their subcomponents include organizational readiness (18 items reflecting extent of any planning, stakeholder buy-in and engagement, change management competencies), technology (16 items reflecting extent to which technology types and features have been discussed/examined, technology infrastructure, any plans to acquire technology best suited to access need,), regulatory and policy (12 items regarding what policies are in place for technology security, patient protection and data security, sharing and organization and other regulatory topics), financing and reimbursement (9 items measuring what investigation has been done with respect to reimbursement, have cost/benefit analyses been conducted, considerations for insurance payor for technology services), clinical (7 items measuring how incorporating technology aligns with organizational mission, beliefs, service goals, cultural responsiveness and referral practices), and workforce (6 items measuring workforce training, motivation, comfort level and experience in using technology for service delivery and practice). Response options are “Don’t Know/Not Applicable” (0), “No, never considered” (1), “No, but have considered” (2), “Yes, in progress (3), “Yes, nearly completed (4), and “Yes, in place” (5) for each item. Mean scores were calculated within each of the six domains. After presenting aggregated first timepoint results to the JTC, stakeholders expressed that it would be helpful to have distinct response options for “Don’t Know” and “Not Applicable”; the TCAT was revised to split into two response options for timepoints two and three such that “Don’t Know” was assigned a (0) and “Not Applicable” was considered missing data. Response rates varied from 50.0 to 69.2% of stakeholders at any given timepoint.

### Qualitative — meeting notes.

De-identified monthly general (i.e., not subcommittee) meeting notes from May 2020 through September 2021 (*N* = 17 meetings) were analyzed. While there was no structured notetaking format, notetakers documented the process and content of conversations. Participating stakeholders were asked to provide feedback on the themes and representative quotes in Table 1 prior to publication; stakeholder feedback suggested that qualitative themes and representative quotes were an accurate reflection of their experience in meetings.

**Table 1:** Qualitative themes and representative quotes from meeting notes

TCAT domain	Meeting minute examples
Organizational readiness	Agencies might not have bandwidth to schedule additional meetings for work groups right now All facing wider concerns/questions about how to use technology [to engage youth/families] Planned for sustainability and steps to keep telehealth going after [the pandemic] Identified gaps in system representation and new stakeholders who should be recruited
Technology	It was evident that community-based providers for system-involved youth were lower on the priority list than clinics serving unhoused persons, because of where the funding comes from; notably, the stakeholder was not sure where the funding came from, signaling a lack of explanation for how the decision was made. As a result, county-level administrators were still working to distribute items like headsets and webcams to providers in January 2021, acknowledging that providers were relying on personal equipment in the meantime Stakeholder 1: There should be endless cell phones for systems-involved youth… Stakeholder 2: … and internet access Some families don’t have access to email to get info about how to get devices Families are generally embracing technology Engagement is more frequent Telehealth did not allow for [seeing] body language and visual cues There is something unique about an in-person relationship that you cannot work around indefinitely This interaction between stakeholders from four different agencies exemplifies this challenge: Stakeholder 1: [We] can only use Zoom to communicate with school district-provided Chromebooks for school purposes; [this is a] barrier for case managers. Google Meet can be used with anyone. Stakeholder 2: Zoom is only for school district personnel and only in community-based organization partnership meeting; this is a continued, currently ongoing issue with legal. Stakeholder 3: “[Our agency] doesn’t support Skype technologically so can’t even dial in to Skype calls that clients and partners may be setting up. Stakeholder 4: “Each agency is using a telehealth platform of their choosing.” One stakeholder described their decision to change from Bluejeans to Zoom because the former “didn’t have all the functionality.” [There’s a] waiting room issue: BlueJeans does not have the waiting room option so when you join, you join whoever is already there
Regulatory and policy	Youth should have a confidential space to participate in their court hearings It's taking so much effort to set everything up, so there's not much time to think of these questions we're asking because it's a luxury to think it through rather than react to it Not sure if youth can participate in court proceedings while in school Physical space limitations at school that would require staff to work around timing/space limits/scheduling
Financing and reimbursement	[We’re] trying to manage [the]economic crisis… [we] submitted budgets to meet cuts from the city… [the city’s] saying they’re not cutting enough In the midst of budget changes, it’s a push/pull with needing to provide more supports and working with partners (e.g., DPH, community-based providers) to expand prevention services… at the same time, [we] need to look at what constitutes CORE services, protect those, and then everything else needs to be considered for [inclusion in the] budget [It’s] hard to feel ready re: financial readiness because unclear where everything is going, how resources will be allocated, how long this will go on, etc Can’t bill for text messages, [which is a] limitation… [we]can’t do a lot of other things without regulations clarified. Need face-to-face contact to be able to make a diagnosis but that can be a barrier right now
Clinical	Growing Latinx population receiving [telehealth] services while African-American population is decreasing in services accessed post-COVID [There is]…still small number of clients where telehealth is not effective (severity of need, maturity levels); still have difficult time reaching higher needs
Workforce	COVID-19 pandemic and civil unrest in response to the killings of Black Americans by the police was affecting work… [it’s] much harder to be having face-to-face contact for work that is relational is challenging while supporting staff who are law enforcement Before COVID: Attorneys could meet with clients before court before but not now; they often meet clients for the first time in court (due to lack of prior access) Difficult to engage with some youth and families. Places where you might normally try to meet clients and families (e.g., parks) have been closed OR the neighborhood does not feel safe. Families are not social distancing or wearing facemasks Now that there's more free time from things like reduced travel time, we see more engagement from staff, like in meetings [There are] computers in each unit [of juvenile hall], parents can come to unit or call from home, calls are [available from] within the hall using Microsoft Teams. Also use for release planning, attorney visits, community orgs to contact kids

### Data analysis

Quantitative data were analyzed using SPSS 27.^24^ Mean scores were calculated for all six domains and then stratified by system. Qualitative data were analyzed via atlasTI.7 in Windows^25^ using Inductive Thematic Analysis methods.^26^ The initial codebook was derived from the six TCAT domains related to incorporating technology into practice and was further refined based on emergent themes identified by two research team members (EH, CO) through preliminary coding of two de-identified meeting notes. This process was repeated until all redundancies were removed and potential themes were identified. The two members of the research team (EH, CO) then consulted with two qualitative experts (ED, JY) on the research team who made minor revisions and provided feedback on the preliminary codebook. Regular meetings between these two researchers (EH, CO) and the qualitative experts (ED, JY) were held to reach consensus and to develop the finalized codebook. Once finalized, the codebook was used with qualitative analysis computer software^25^ for identification of major themes by two members on the research team (EH, KL) with all meeting notes.

## Results

### Quantitative — TCAT domain scores

At timepoint 1, 9 stakeholders from 5 different systems completed the TCAT. Organizational readiness (stakeholder buy-in, planning, competencies) was the area of greatest need (*M* = 2.54, *SD* = 0.69) as compared to regulatory and policy (*M* = 3.27, *SD* = 1.38) and clinical (*M* = 3.14, *SD* = 1.02) domains in which more progress was perceived in incorporating technology into their system.

At timepoint 2, 9 stakeholders from 5 different systems indicated that the technology domain was building capacity, and overall responses indicated 6-month progress in each domain (range = 2.92 to 3.67). Similar to timepoint 1, the regulatory/policy (*M* = 3.58, *SD* = 1.26) and clinical (*M* = 3.67, *SD* = 0.64) domains continued to be rated as having the most capacity with respect to technology use in their respective systems.

At timepoint 3, 6 stakeholders from 5 different systems indicated improved capacity in organizational readiness (*M* = 3.07, *SD* = 0.33), clinical (*M* = 3.46, *SD* = 0.87), and workforce development (*M* = 3.40, *SD* = 0.80) domains. The greatest 6-month capacity progress was in financing/reimbursement, regulatory/policy, and workforce development domains, and small capacity declines in clinical and technology domains.

#### Domain scores by stakeholder system

Stakeholders from 6 systems completed the TCAT at 1 or more timepoints over an 18-month period. Despite system-level continuity in TCAT completion, different participants commonly completed the TCAT at each time point (e.g., participant A for system A at timepoint 1; participant B for system A at timepoint 2). Results thus represent system-level snapshots provided by different stakeholders within the same system. Over 18 months, most systems reported building technology capacity, but progress was not always sustained by timepoint 3. For example, educational stakeholder’s responses reflected progress in technology, regulatory/policy, clinical, and workforce domains from timepoint 1 to timepoint 2 but declines in capacity by timepoint 3. In contrast, probation stakeholders reported incremental progress across all timepoints and domains except for financing and reimbursement.

### Qualitative results

#### Organizational readiness

Rapid response to COVID-19 and the shelter-in-place ordinance led stakeholders to prioritize organizational readiness to integrate technology into practice as the primary JTC focus. Emergent themes in this area included initial changes to the JTC’s scope of work in response to system needs and bandwidth, as well as recruitment of new JTC stakeholders.

At the first meeting after the onset of COVID-19 in May 2020, systems faced an unprecedented challenge to serving all youth and families under their jurisdiction. A prolonged discussion about the JTC purpose and vision led to a shift from initial focus of consultations on specific tech-based research projects to creating a protected space to share information and learn from each other as they faced wider concerns and questions about how to use technology to engage youth/families in the face of a pandemic. Stakeholders also decided to expand the scope of work to focus on immediate need of remote service provision while considering longer term technology sustainability. JTC members identified new stakeholders with needed expertise (e.g., directors of community-based clinical service programs) to invite to the JTC to fill gaps in stakeholder representation due to this expanded scope.

#### Technology

Given the explicit mission of the JTC, technology was a frequent topic of discussion. Prominent themes included (1) facilitators and barriers to building technology infrastructure, (2) challenges staff faced when using technology to interface with families and across systems, and (3) materials and training to improve comfort and effectiveness of technology.

Stakeholders identified overburdened IT departments as a barrier that prevented timely distribution of technology tools to clients and staff. Stakeholders were consistently in agreement with prioritizing technology infrastructure for clients over staff; this was exemplified through an interaction between stakeholders from two different systems centered on making cell phones and internet access readily available to all system-impacted youth and families at the onset of the COVID-19 pandemic. The public school district distributed technology to students who lacked equipment, but assumptions about families’ technology literacy, such as requiring an email address to request devices, led to delays in technology distribution to families who did not have one. Once schools distributed Chromebook laptops, WIFI hotspots reached students and laptops were enabled for non-school-related purposes, participating JTC systems were able to re-focus their efforts on behavioral health services access. However, this re-focus then highlighted an important infrastructural sub-theme that system-siloed selection of technology platforms led to multiple technology systems and platforms being used, preventing data sharing and collaboration needed to coordinate timely and relevant behavioral health care access. Stakeholders were familiar with the specific platforms’ compatibility and functionality for different purposes, with some organizations blocking or not supporting the use of some platforms that were preferred by clients and other partnering organizations (e.g., Skype being familiar and used by clients but not allowable for use by service systems due to privacy mandates). Some systems recognized their initial selection of technology platforms did not meet their needs and pivoted (e.g., switch from BlueJeans to use Zoom’s breakout room function). One stakeholder described their internal decision-making process as first identifying what their own system needed and then selecting a technology platform. Notably, this process did not include soliciting feedback or assessing inter-operability with other system partners or clients/families.

Staff challenges in providing telehealth services included ergonomic issues, lack of privacy/confidentiality, changes to work routines, lower tech literacy, inability to capture necessary signatures for documentation (e.g., consent, release of information), and challenges using non-English language interpretation services. Stakeholders noted telehealth limitations around being able to rely on body language and visual cues and developing in-person relationships. Most hoped to implement a hybrid (i.e., remote via technology and in-person) model for service delivery for the long-term.

Stakeholders provided examples of their systems’ efforts in this area. For example, the school district coordinated volunteers who provided individualized technical support to set up technology as well as created Zoom training videos for youth and families. Stakeholders noted that as a result, families were embracing technology with more frequent engagement, thereby improving service access. Stakeholders noted, however, that such benefits were not universal and that they experienced differences in technology utilization and engagement according to cultural factors and acuity of youth behavioral health needs.

#### Regulatory and policy

JTC stakeholder discussion was least in this domain but when raised centered on a main theme of highly underdeveloped regulatory and policy guidance around telehealth privacy and confidentiality, particularly for legal proceedings. Stakeholders recognized that youth should be able to participate in legal proceedings remotely from home, school, and other environments, but there were no policies to guide where and how youth could participate in such proceedings with privacy and confidentiality. Clinician and school stakeholders shared similar concerns around tele-behavioral healthcare privacy and confidentiality policies. Stakeholders acknowledged that telehealth policy development was more reactive than proactive and developing organizational policies ahead of time was perceived as a luxury that stakeholders did not have in the face of the COVID-19 changing landscape.

#### Financing and reimbursement

Prominent financing themes included (1) budgetary challenges and the impact on behavioral health services access (2) funding sources, and (3) tele-behavioral health services reimbursement procedures.

Stakeholders highlighted budgetary challenges related to the COVID-19 pandemic and ensuing economic crisis affecting agencies serving system-impacted youth and families at a time when demand for behavioral health services increased. Amidst cuts, stakeholders focused on deciding what currently available behavioral health services were core and identified expansion of prevention services as a gap to be addressed.

Stakeholders raised questioned the feasibility of new JTC project ideas without resources. The engagement subcommittee proposed building an automated database to improve efficiency for behavioral health provider referrals; however, lack of imminent person and financial resources to develop, build, and sustain such database were raised as key barriers to this being a viable project pursuit. The research team and JTC stakeholders discussed funding opportunities, as they arose, to support project ideas co-developed with JTC stakeholders. Overall, lack of funding was noted as an ongoing concern for existing service delivery as well as a barrier to expansion of preventive services, the development of new technology-based projects to improve service access and JTC scope expansion.

MediCal/Medicaid funding for behavioral health services were discussed particularly related to regulations allowing for reimbursement for telehealth services after the COVID-19 pandemic onset. Child welfare and behavioral health systems described a need for sustained telehealth reimbursement and lack of reimbursement for certain modalities like text messaging, which reduced optimism about sustainability of technology interventions.

#### Clinical

JTC stakeholder discussion in this domain centered primarily on the theme of improving access to existing behavioral health services. In July 2020, just after the COVID-19 pandemic onset, stakeholders noted initial success in improving services access for those without technology limitations but that a subset of families with more severe behavioral health needs did not benefit. In November 2020, behavioral health service data for youth was presented highlighting trends in service access over the first 6 months of the pandemic. Data reflected a 6% reduction in new services opened from March to June 2020 for justice-impacted youth followed by a larger 64% reduction from June to September 2020, which was attributed to delays in case processing from the justice system to behavioral health providers. Additionally, less than 5% of justice-impacted youth were receiving substance use services as of September 2020, which was attributed in part to the fact that there was limited workforce capacity to provide dual-diagnosis (i.e., substance use and mental health) treatment for justice-involved youth. Stakeholders brainstormed on how to increase the availability of those services, which led to a TCAT Capacity Strengthening Plan in the Initial Access subcommittee, to develop a dual-diagnosis training and consultation curriculum delivered via telehealth (Project ECHO JJBH).

#### Workforce

Front-line staff were frequently discussed across systems, especially how they were coping with changes in working remotely after the onset of the COVID-19 pandemic. The most prominent discussion theme was related to impediments and successes staff faced in providing direct service.

Stakeholders described frustration with providing services when required through COVID-19 health mandates to work remotely. In June 2020, stakeholders discussed how the COVID-19 pandemic and civil unrest in response to the killings of Black Americans by the police presented challenges for front-line staff; these were exacerbated by working remotely and the inability to physically interface and connect with youth and families. Legal stakeholders also described how remote work impeded workflow with detained youth clients, particularly when meeting just prior to court hearings and ability to have private conversations given the physical environment (e.g., accessible phones are next to the guard kiosk*).* Additionally, some technology platforms lacked the functionality necessary to mimic in-person (i.e., pre-COVID) court proceedings. For example, technology used for remote court proceedings did not include separate private meeting capability options beforehand, making it harder for attorneys and others to interface with youth clients. Other barriers cited included personal safety and concerns that youth may not have been adhering to measures designed to limit the spread of COVID-19, thereby impairing staff’s ability to identify behavioral health needs safely and accurately for treatment referral purposes.

Stakeholders frequently mentioned how staff actively problem-solved to overcome the aforementioned barriers. Probation and behavioral health stakeholders described positive aspects of remote work such as reduced travel time and increased client engagement. Probation noted that a combination of technology infrastructure, specific software, and allocation of physical space in juvenile hall were essential to reducing social isolation of detained youth; this solution facilitated interaction with family members and increased access to providers and defense attorneys.

## Discussion

The JTC has fostered cross-system collaboration among stakeholders tasked with meeting the behavioral health needs of youth impacted by the juvenile justice and child welfare systems. The JTC’s mission to leverage technology to increase services access was timely at the onset of COVID-19 to rapidly develop remote service delivery capacity. Academic research and judicial stakeholder co-leadership, a community participatory research approach, logistical adaptability and flexibility, and balanced cross-sector stakeholder recruitment were key to the evolution, growth, and sustainment of the JTC.

Quantitative results indicated that factors influencing readiness to implement telehealth services were dynamic across systems and time; for example, organizational readiness to use technology to that end was initially low but generally improved or stayed the same over time. Overall, most systems reported building technology-related capacity within each domain over the first 12 months, but by 18 months capacity-building progress stalled or marginally declined in most systems and within most domains. These findings might reflect overall higher rates of technology-use related burnout by staff^27^ due to remote work since the onset of COVID-19.^28,29^ A lack of sustainable resources to maintain technology infrastructure originally provided for immediate COVID-19 relief may have also contributed.

The technology domain was an obvious focal area for discussion given the JTC mission. Many of the challenges raised were consistent with other studies of technology-delivered services to youth and families,^30^ including barriers to staff delivering telehealth appropriately (e.g., training, privacy, ergonomics, device, and Wi-Fi availability for staff and youth), overburdened system information technology (IT) departments, and clear inequities in which youth and families were able to access and engage in technological interventions. Cross-system discussions on these topics led to sharing and/or expansion of resources (e.g., changing IT settings on school-provided laptops allowing youth to attend court hearings through school-provided laptops). While quantitative results suggest the Regulatory and Policy capacity to be the most advanced domain, qualitative data show it was discussed the least. This perhaps reflects a stakeholder preference to raise significant concerns and challenges (vs. successes) while collaborating across systems; meeting discussions reflected multiple systems reporting requirements to imminently address regulatory and policy issues when they were reacting to specific situations versus proactively developing organizational policies around technology use.

The JTC provided unique opportunities for systems who do not ordinarily discuss workforce challenges together to do so and problem-solve on ways in which technology could help address shared and unique workforce challenges across systems. For example, funding and budgetary challenges were often highlighted as a barrier to leveraging technology to increase access to care. Questions were raised about which system’s budget would be responsible or the “home” for technology interventions and budgetary decisions in the context of cross-system collaborative projects such as those proposed by the JTC.

### Limitations and future considerations

A small sample size, inconsistent system respondents across timepoints, and missing data must be taken into consideration for quantitative findings. These limitations precluded rigorous statistical analysis of system-level differences; however, descriptive TCAT results still provided a useful initial snapshot of progress toward incorporating technology into practice. Unfortunately, the sample was too small to assess psychometric properties of the TCAT and there are no published papers with these data. Despite limitations, aggregate data presented back to stakeholders provided a platform for rich and useful discussions of challenges and successes each system faced in integrating technology into practice. This study also focused on a single county jurisdiction and requires replication. Key recommendations for successful replication include using existing cross-system models and structures to build the JTC; partnering with academic researchers who can bring unique logistical, technology, and financial resources to the JTC; and identifying existing public health initiatives that can be integrated with technology-informed health equity efforts for system-impacted youth. Lastly, while the TCAT and Cascade frameworks were used to guide the JTC development and process, this study did not include an empirically supported implementation science framework that, for example, would guide measurement of cross-collaborative strategies, organizational change processes, and relational factors. Future research on public health-justice technology collaboratives should also incorporate measurement of multiple intra- and interorganizational change factors to develop and empirically test general organizational and cross-collaborative strategies and approaches. Nevertheless, the TCAT and Cascade frameworks offered ideal starting points for the JTC given the mission to address the intersection of technology and behavioral health services delivery and to build systems’ capacity to improve services delivery through utilization of technology.

## Implications for Behavioral Health

The JTC began with the goal of focusing on leveraging one type of technology (i.e., text messaging) across public health and justice systems to improve system-impacted youth substance use services access, engagement, and retention. The COVID-19 pandemic and stakeholders’ desire to concurrently carry out multiple projects using multiple technologies required a rapid expansion of the JTC and diverse cross-collaborative approaches and capacity strengthening plans. Bringing systems together to identify cross-system priorities, strategies, and plans to use technology to increase behavioral health care access for system-impacted youth revealed multiple gaps in providing necessary coordinated care and a critical need to identify novel, rapid solutions to fill those gaps. The JTC serves as an innovative model for behavioral health, diversion, legal, child welfare, school, and juvenile probation systems to come together to develop, implement, and research outcomes of structural interventions that aim to advance behavioral health equity and services access through technology for system-impacted youth.
